# Bis(4-amino­benzene­sulfonato-κ*O*)bis­(propane-1,3-diamine-κ^2^ *N*,*N*′)copper(II) dihydrate

**DOI:** 10.1107/S1600536809049769

**Published:** 2009-11-25

**Authors:** Ke-Juan Zhang, Xiang-Gao Meng, Xiu-Ling Li

**Affiliations:** a School of Chemistry and Chemical Engineering, Xuzhou Normal University, Xuzhou, Jiangsu 221116, People’s Republic of China; b Key Laboratory of Pesticides & Chemical Biology, College of Chemistry, Central China Normal University, Wuhan, Hubei 430079, People’s Republic of China

## Abstract

In the title compound, [Cu(C_3_H_10_N_2_)_2_(C_6_H_6_NO_3_S)_2_]·2H_2_O, the Cu^II^ atom lies on an inversion center and is hexa­coordinated by four N atoms from two 1,3-diamino­propane ligands and two O atoms from two 4-amino­benzene­sulfonate ligands in a *trans* arrangement, displaying a distorted and axially elongated octa­hedral coordination geometry, with the O atoms at the axial positions. A three-dimensional network is formed in the crystal structure through O—H⋯O, N—H⋯O and N—H⋯N hydrogen bonds.

## Related literature

For general background to crystal engineering based on metal and organic building blocks, see: Evans & Lin (2002 ▶); Li *et al.* (2003 ▶, 2004 ▶). For related structures, see: Kim & Lee (2002 ▶); Sundberg *et al.* (2001 ▶); Sundberg & Sillanpää (1993 ▶); Sundberg & Uggla (1997 ▶); Wang *et al.* (2002 ▶). For the synthesis, see: Gunderman *et al.* (1996 ▶).
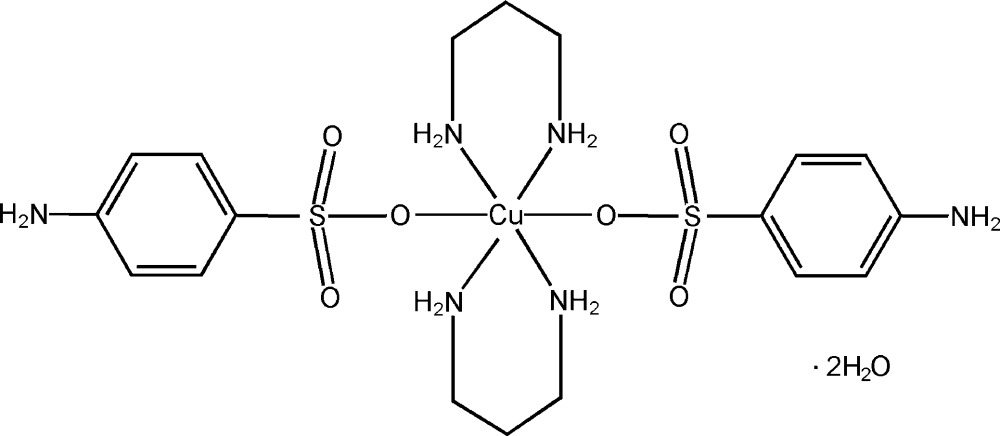


## Experimental

### 

#### Crystal data

[Cu(C_3_H_10_N_2_)_2_(C_6_H_6_NO_3_S)_2_]·2H_2_O
*M* *_r_* = 592.19
Monoclinic, 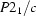

*a* = 9.5171 (1) Å
*b* = 10.3875 (4) Å
*c* = 13.1646 (5) Å
β = 101.256 (2)°
*V* = 1276.40 (7) Å^3^
*Z* = 2
Mo *K*α radiation
μ = 1.07 mm^−1^
*T* = 293 K
0.48 × 0.20 × 0.18 mm

#### Data collection

Siemens SMART 1000 CCD diffractometer
Absorption correction: multi-scan (*SADABS*; Sheldrick, 1996 ▶) *T*_min_ = 0.627, *T*_max_ = 0.830
3629 measured reflections
2230 independent reflections
1889 reflections with *I* > 2σ(*I*)
*R*_int_ = 0.024

#### Refinement

*R*[*F* ^2^ > 2σ(*F* ^2^)] = 0.048
*wR*(*F* ^2^) = 0.132
*S* = 1.09
2230 reflections
161 parameters
3 restraints
H-atom parameters constrained
Δρ_max_ = 0.48 e Å^−3^
Δρ_min_ = −0.41 e Å^−3^

### 

Data collection: *SMART* (Siemens, 1996 ▶); cell refinement: *SAINT* (Siemens, 1996 ▶); data reduction: *SAINT*; program(s) used to solve structure: *SHELXS97* (Sheldrick, 2008 ▶); program(s) used to refine structure: *SHELXL97* (Sheldrick, 2008 ▶); molecular graphics: *SHELXTL* (Sheldrick, 2008 ▶); software used to prepare material for publication: *SHELXTL*.

## Supplementary Material

Crystal structure: contains datablocks I, global. DOI: 10.1107/S1600536809049769/hy2252sup1.cif

Structure factors: contains datablocks I. DOI: 10.1107/S1600536809049769/hy2252Isup2.hkl

Additional supplementary materials:  crystallographic information; 3D view; checkCIF report

## Figures and Tables

**Table 1:** Selected bond lengths (Å)

Cu—N1	2.038 (3)
Cu—N2	2.029 (3)
Cu—O1	2.589 (3)

**Table 2:** Hydrogen-bond geometry (Å, °)

*D*—H⋯*A*	*D*—H	H⋯*A*	*D*⋯*A*	*D*—H⋯*A*
O1*W*—H1*WA*⋯O2	0.85	1.90	2.651 (5)	146
O1*W*—H1*WB*⋯O3^i^	0.85	2.16	2.969 (8)	160
N1—H1*A*⋯N3^ii^	0.90	2.46	3.250 (5)	147
N1—H1*B*⋯O3^iii^	0.90	2.39	3.243 (4)	158
N2—H2*A*⋯O3^iv^	0.90	2.42	3.183 (4)	143
N2—H2*B*⋯O1*W* ^v^	0.90	2.13	3.025 (5)	177
N3—H3*D*⋯O1*W* ^vi^	0.86	2.69	3.337 (6)	133
N3—H3*C*⋯O1^vii^	0.86	2.46	3.248 (5)	153

Symmetry codes: (i) 
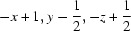
; (ii) 
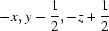
; (iii) 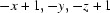; (iv) 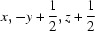; (v) 
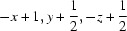
; (vi) 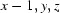; (vii) 
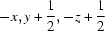
.

**Fig. 1.**
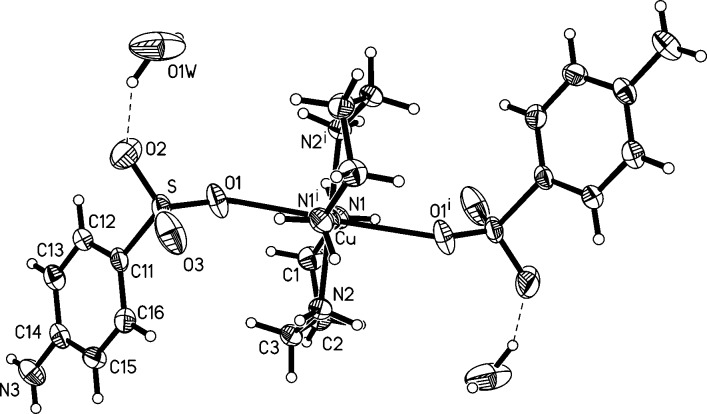
Molecular structure of the title compound. Displacement ellipsoids are drawn at the 30% probability level. Dashed lines denote hydrogen bonds. [Symmetry code: (i) 1 - x, -y, 1 - z.]

**Fig. 2.**
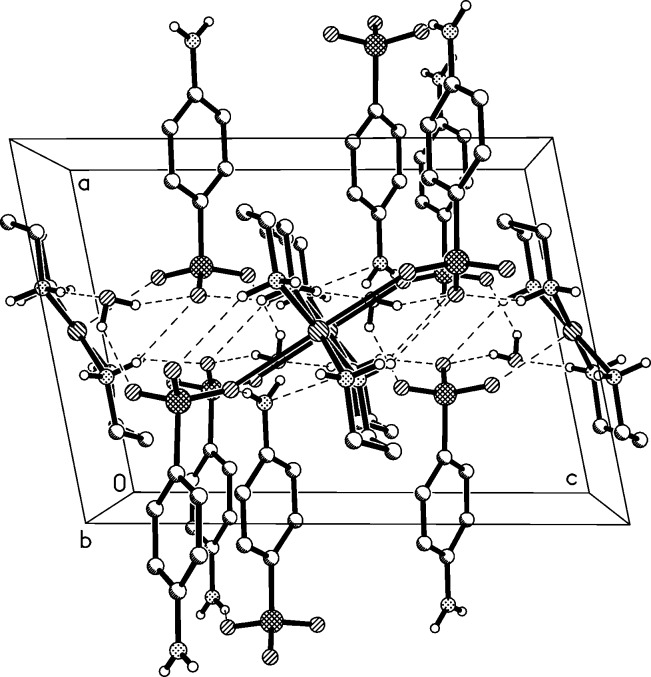
The packing diagram of the title compound viewed along the b axis. H atoms not involved in hydrogen bonds (dashed lines) are omitted for clarity.

## supplementary crystallographic information

### Comment

Crystal engineering based on metal and organic building blocks has been rapidly developed in recent years owing to their novel and diverse topologies and potential applications in catalysis and host–guest chemistry (Evans & Lin, 2002). Covalent bonds and hydrogen bonds have been demonstrated to be two important interactions in constructing metal-containing supramolecular frameworks, and they have brought forth a great variety of novel frameworks with fascinating structural motifs (Li *et al.*, 2003, 2004). 1,3-Diaminopropane (tn) ligand behaves as a strong chelatator in its metal complexes due to the formation of a stable six-membered ring. At the same time, it is a good H-bond donor due to the existence of amino groups (Sundberg *et al.*, 2001). The crystal egineering of tn and carboxylate ligands has been studied in detail (Sundberg *et al.*, 2001), but supramolecular chemistry of tn and 4-aminobenzenesulfonate (4-*ABS*) ligand is still not explored to that extent (Wang *et al.*, 2002). 4-*ABS* can act as a bridging or a terminal ligand in its metal complexes. On the other hand, studies on the coordination and supramolecular chemistry of 4-*ABS* have showed that it is a good H-bond acceptor and can form strong H-bonds due to its three O atoms and one N atom (Kim & Lee, 2002; Wang *et al.*, 2002). In view of their excellent coordination capability and good H-bond donor or acceptor nature, we employed tn and 4-*ABS* as mixed organic building blocks to construct supramolecular networks in an expectation that these ligands may generate hydrogen bonding and/or covalent interactions with transition metal ions in the assembly process. Herein, we report the synthesis and structure of the title compound.

As shown in Fig. 1, the Cu^II^ atom lies on an inversion center and is octahedrally coordinated by four N atoms from two tn ligands and two O atoms from two 4-*ABS* ligands in a *trans* arrangement. The coordination polyhedron of the Cu^II^ ion can be described as axially elongated octahedral, with the O atoms at the axial positions. The tn ligand shows chelating coordination behavior and displays a chair conformation in the equatorial direction. This kind of coordination mode was also found in the similar complexes (Sundberg *et al.*, 2001; Sundberg & Sillanpää, 1993; Sundberg & Uggla, 1997). The axial Cu—O distance is 2.589 (3) Å, indicating a weak coordination. The equatorial Cu—N1 and Cu—N2 bond lengths are 2.038 (3) and 2.029 (3) Å, respectively, which are much shorter than the axial Cu—O distance and very similar to those in the previously reported *trans*-bis(4-methylbenzenesulfonato)bis(1,3-diaminopropane)copper(II) (Sundberg & Sillanpää, 1993). The tn molecule forms a six-membered chelate ring with asymmetric Cu—N1—C1 and Cu—N2—C3 angles of 122.7 (2) and 119.9 (2)°. A plausible explanation for the deviations described above may be attributed to the asymmetric hydrogen bonding with respect to the chelate ring. The complex molecules are linked into a two-dimensional layer through hydrogen bonds between the uncoordinated water, the sulfonate group and the amino groups of the tn ligand. The layers are further connected into a three-dimensional network through hydrogen bonds between the amino groups and the sulfonate groups of neighboring 4-*ABS* ligands (Fig. 2).

### Experimental

Diaquabis(4-aminobenzenesulfonato)copper(II) dihydrate was synthesized according to the literature (Gunderman *et al.*, 1996). 1,3-Diaminopropane (0.35 g, 4.72 mmol) in 10 ml water was dropped slowly into the stirred diaquabis(4-aminobenzenesulfonato)copper(II) dihydrate (1.12 g, 2.33 mmol) solution in 20 ml water. The mixed solution was kept stirring at room temperature for 30 min. After filtration, the filtrate was left to evaporate in air. After a few days, blue crystals of the title compound suitable for X-ray study were obtained (yield 0.70 g, 51%).

### Refinement

H atoms bonded to C atoms or N atoms were positioned geometrically and refined as riding atoms, with C—H = 0.93 (aromatic) and 0.97 (CH_2_) Å and N—H = 0.90 and 0.86 Å and with *U*_iso_(H) = 1.2*U*_eq_(C,*N*). Water H atoms were located in a difference Fourier map and refined as riding, with O—H = 0.85 Å and with *U*_iso_(H) =1.5*U*_eq_(O).

### Crystal data

**Table:**

[Cu(C_3_H_10_N_2_)_2_(C_6_H_6_NO_3_S)_2_]·2H_2_O	*F*(000) = 622
*M**_r_* = 592.19	*D*_x_ = 1.541 Mg m^−^^3^
Monoclinic, *P*2_1_/*c*	Mo *K*α radiation, λ = 0.71073 Å
Hall symbol: -P 2ybc	Cell parameters from 2312 reflections
*a* = 9.5171 (1) Å	θ = 2.2–25.1°
*b* = 10.3875 (4) Å	µ = 1.07 mm^−^^1^
*c* = 13.1646 (5) Å	*T* = 293 K
β = 101.256 (2)°	Prism, blue
*V* = 1276.40 (7) Å^3^	0.48 × 0.20 × 0.18 mm
*Z* = 2	

### Data collection

**Table:**

Siemens SMART 1000 CCD diffractometer	2230 independent reflections
Radiation source: fine-focus sealed tube	1889 reflections with *I* > 2σ(*I*)
graphite	*R*_int_ = 0.024
φ and ω scans	θ_max_ = 25.1°, θ_min_ = 2.2°
Absorption correction: multi-scan (*SADABS*; Sheldrick, 1996)	*h* = −9→11
*T*_min_ = 0.627, *T*_max_ = 0.830	*k* = −7→12
3629 measured reflections	*l* = −15→10

### Refinement

**Table:**

Refinement on *F*^2^	Secondary atom site location: difference Fourier map
Least-squares matrix: full	Hydrogen site location: inferred from neighbouring sites
*R*[*F*^2^ > 2σ(*F*^2^)] = 0.048	H-atom parameters constrained
*wR*(*F*^2^) = 0.132	*w* = 1/[σ^2^(*F*_o_^2^) + (0.0669*P*)^2^ + 1.3129*P*] where *P* = (*F*_o_^2^ + 2*F*_c_^2^)/3
*S* = 1.09	(Δ/σ)_max_ < 0.001
2230 reflections	Δρ_max_ = 0.48 e Å^−^^3^
161 parameters	Δρ_min_ = −0.41 e Å^−^^3^
3 restraints	Extinction correction: *SHELXL97* (Sheldrick, 2008), Fc^*^=kFc[1+0.001xFc^2^λ^3^/sin(2θ)]^-1/4^
Primary atom site location: structure-invariant direct methods	Extinction coefficient: 0.054 (4)

### Fractional atomic coordinates and isotropic or equivalent isotropic displacement parameters (Å^2^)

**Table:**

	*x*	*y*	*z*	*U*_iso_*/*U*_eq_	
Cu	0.5000	0.0000	0.5000	0.0387 (3)	
N1	0.3723 (3)	−0.1305 (3)	0.5539 (2)	0.0470 (7)	
H1A	0.3788	−0.2043	0.5193	0.056*	
H1B	0.4125	−0.1457	0.6205	0.056*	
N2	0.3801 (3)	0.1490 (3)	0.5351 (2)	0.0457 (7)	
H2A	0.4171	0.1731	0.6005	0.055*	
H2B	0.3929	0.2153	0.4938	0.055*	
C1	0.2183 (4)	−0.1079 (4)	0.5506 (3)	0.0565 (10)	
H1C	0.1820	−0.1742	0.5905	0.068*	
H1D	0.1669	−0.1143	0.4796	0.068*	
C2	0.1902 (4)	0.0226 (4)	0.5934 (3)	0.0537 (10)	
H2C	0.0902	0.0277	0.5990	0.064*	
H2D	0.2476	0.0314	0.6625	0.064*	
C3	0.2240 (4)	0.1327 (4)	0.5273 (3)	0.0519 (9)	
H3A	0.1794	0.1169	0.4557	0.062*	
H3B	0.1840	0.2116	0.5491	0.062*	
O1W	0.5872 (4)	−0.1325 (5)	0.1104 (5)	0.147 (2)	
H1WA	0.5115	−0.0876	0.0934	0.221*	
H1WB	0.5700	−0.1840	0.1563	0.221*	
S	0.31964 (10)	0.04586 (13)	0.21957 (7)	0.0585 (4)	
O1	0.3474 (3)	−0.0243 (3)	0.3153 (3)	0.0735 (10)	
O2	0.3401 (4)	−0.0303 (6)	0.1333 (3)	0.156 (3)	
O3	0.4010 (3)	0.1646 (4)	0.2294 (3)	0.0936 (13)	
C11	0.1360 (4)	0.0876 (4)	0.1932 (2)	0.0435 (8)	
C12	0.0384 (4)	0.0061 (4)	0.1338 (3)	0.0488 (9)	
H12A	0.0702	−0.0675	0.1050	0.059*	
C13	−0.1068 (4)	0.0337 (4)	0.1168 (3)	0.0527 (10)	
H13A	−0.1717	−0.0220	0.0770	0.063*	
C14	−0.1565 (4)	0.1431 (4)	0.1584 (3)	0.0483 (9)	
C15	−0.0568 (4)	0.2261 (4)	0.2159 (3)	0.0523 (9)	
H15A	−0.0880	0.3016	0.2424	0.063*	
C16	0.0875 (4)	0.1982 (4)	0.2342 (3)	0.0493 (9)	
H16A	0.1525	0.2538	0.2741	0.059*	
N3	−0.3017 (3)	0.1723 (4)	0.1411 (3)	0.0680 (10)	
H3D	−0.3627	0.1219	0.1039	0.082*	
H3C	−0.3305	0.2407	0.1676	0.082*	

### Atomic displacement parameters (Å^2^)

**Table:**

	*U*^11^	*U*^22^	*U*^33^	*U*^12^	*U*^13^	*U*^23^
Cu	0.0294 (4)	0.0433 (4)	0.0434 (4)	0.0037 (2)	0.0068 (2)	0.0027 (2)
N1	0.0377 (16)	0.0488 (17)	0.0541 (18)	0.0005 (13)	0.0079 (13)	0.0047 (14)
N2	0.0406 (16)	0.0493 (17)	0.0478 (17)	0.0061 (13)	0.0103 (13)	−0.0016 (14)
C1	0.0385 (19)	0.064 (2)	0.069 (3)	−0.0065 (18)	0.0147 (18)	−0.005 (2)
C2	0.039 (2)	0.076 (3)	0.049 (2)	0.0008 (18)	0.0162 (17)	−0.0048 (19)
C3	0.0358 (19)	0.068 (3)	0.051 (2)	0.0141 (17)	0.0058 (16)	−0.0058 (19)
O1W	0.061 (2)	0.130 (4)	0.254 (6)	−0.018 (2)	0.038 (3)	−0.109 (4)
S	0.0392 (5)	0.0932 (8)	0.0388 (5)	0.0172 (5)	−0.0031 (4)	0.0027 (5)
O1	0.0558 (18)	0.081 (2)	0.070 (2)	0.0005 (15)	−0.0221 (15)	0.0234 (16)
O2	0.067 (3)	0.294 (7)	0.091 (3)	0.084 (3)	−0.025 (2)	−0.090 (4)
O3	0.0430 (16)	0.125 (3)	0.109 (3)	−0.0080 (18)	0.0046 (17)	0.051 (2)
C11	0.0385 (18)	0.057 (2)	0.0331 (17)	0.0085 (16)	0.0022 (13)	0.0113 (15)
C12	0.049 (2)	0.051 (2)	0.042 (2)	0.0118 (16)	0.0005 (16)	0.0030 (16)
C13	0.044 (2)	0.053 (2)	0.056 (2)	−0.0013 (17)	−0.0026 (17)	0.0072 (18)
C14	0.0387 (19)	0.058 (2)	0.048 (2)	0.0050 (17)	0.0075 (15)	0.0182 (17)
C15	0.054 (2)	0.052 (2)	0.052 (2)	0.0129 (18)	0.0123 (17)	0.0032 (18)
C16	0.050 (2)	0.055 (2)	0.0408 (19)	0.0000 (17)	0.0035 (15)	0.0011 (16)
N3	0.0404 (18)	0.075 (2)	0.087 (3)	0.0107 (17)	0.0085 (17)	0.012 (2)

### Geometric parameters (Å, °)

**Table:**

Cu—N1	2.038 (3)	O1W—H1WB	0.85
Cu—N2	2.029 (3)	S—O2	1.428 (4)
Cu—O1	2.589 (3)	S—O1	1.435 (3)
N1—C1	1.476 (4)	S—O3	1.448 (4)
N1—H1A	0.9000	S—C11	1.768 (3)
N1—H1B	0.9000	C11—C12	1.381 (5)
N2—C3	1.479 (4)	C11—C16	1.386 (5)
N2—H2A	0.9000	C12—C13	1.386 (6)
N2—H2B	0.9000	C12—H12A	0.9300
C1—C2	1.512 (5)	C13—C14	1.384 (6)
C1—H1C	0.9700	C13—H13A	0.9300
C1—H1D	0.9700	C14—N3	1.389 (5)
C2—C3	1.509 (6)	C14—C15	1.391 (6)
C2—H2C	0.9700	C15—C16	1.378 (5)
C2—H2D	0.9700	C15—H15A	0.9300
C3—H3A	0.9700	C16—H16A	0.9300
C3—H3B	0.9700	N3—H3D	0.8600
O1W—H1WA	0.85	N3—H3C	0.8600
			
N2—Cu—N2^i^	180.0	C1—C2—H2D	109.0
N2—Cu—N1	91.61 (13)	H2C—C2—H2D	107.8
N2^i^—Cu—N1	88.40 (13)	N2—C3—C2	111.8 (3)
N2—Cu—N1^i^	88.40 (13)	N2—C3—H3A	109.3
N2^i^—Cu—N1^i^	91.60 (13)	C2—C3—H3A	109.3
N1—Cu—N1^i^	180.0	N2—C3—H3B	109.3
N2—Cu—O1^i^	87.08 (11)	C2—C3—H3B	109.3
N2^i^—Cu—O1^i^	92.92 (11)	H3A—C3—H3B	107.9
N1—Cu—O1^i^	90.08 (11)	H1WA—O1W—H1WB	105.1
N1^i^—Cu—O1^i^	89.92 (11)	O2—S—O1	112.8 (3)
N2—Cu—O1	92.92 (11)	O2—S—O3	112.9 (3)
N2^i^—Cu—O1	87.08 (12)	O1—S—O3	110.5 (2)
N1—Cu—O1	89.92 (11)	O2—S—C11	105.22 (19)
N1^i^—Cu—O1	90.08 (11)	O1—S—C11	107.59 (18)
O1^i^—Cu—O1	180.0	O3—S—C11	107.44 (19)
C1—N1—Cu	122.7 (2)	S—O1—Cu	138.47 (19)
C1—N1—H1A	106.7	C12—C11—C16	119.4 (3)
Cu—N1—H1A	106.7	C12—C11—S	119.4 (3)
C1—N1—H1B	106.7	C16—C11—S	121.2 (3)
Cu—N1—H1B	106.7	C11—C12—C13	120.2 (4)
H1A—N1—H1B	106.6	C11—C12—H12A	119.9
C3—N2—Cu	119.9 (2)	C13—C12—H12A	119.9
C3—N2—H2A	107.3	C14—C13—C12	120.8 (4)
Cu—N2—H2A	107.3	C14—C13—H13A	119.6
C3—N2—H2B	107.3	C12—C13—H13A	119.6
Cu—N2—H2B	107.3	C13—C14—N3	121.3 (4)
H2A—N2—H2B	106.9	C13—C14—C15	118.3 (3)
N1—C1—C2	112.3 (3)	N3—C14—C15	120.3 (4)
N1—C1—H1C	109.1	C16—C15—C14	121.0 (4)
C2—C1—H1C	109.1	C16—C15—H15A	119.5
N1—C1—H1D	109.1	C14—C15—H15A	119.5
C2—C1—H1D	109.1	C15—C16—C11	120.1 (4)
H1C—C1—H1D	107.9	C15—C16—H16A	119.9
C3—C2—C1	113.1 (3)	C11—C16—H16A	119.9
C3—C2—H2C	109.0	C14—N3—H3D	120.0
C1—C2—H2C	109.0	C14—N3—H3C	120.0
C3—C2—H2D	109.0	H3D—N3—H3C	120.0

Symmetry codes: (i) −*x*+1, −*y*, −*z*+1.

### Hydrogen-bond geometry (Å, °)

**Table:**

*D*—H···*A*	*D*—H	H···*A*	*D*···*A*	*D*—H···*A*
O1W—H1WA···O2	0.85	1.90	2.651 (5)	146
O1W—H1WB···O3^ii^	0.85	2.16	2.969 (8)	160
N1—H1A···N3^iii^	0.90	2.46	3.250 (5)	147
N1—H1B···O3^i^	0.90	2.39	3.243 (4)	158
N2—H2A···O3^iv^	0.90	2.42	3.183 (4)	143
N2—H2B···O1W^v^	0.90	2.13	3.025 (5)	177
N3—H3D···O1W^vi^	0.86	2.69	3.337 (6)	133
N3—H3C···O1^vii^	0.86	2.46	3.248 (5)	153

Symmetry codes: (ii) −*x*+1, *y*−1/2, −*z*+1/2; (iii) −*x*, *y*−1/2, −*z*+1/2; (i) −*x*+1, −*y*, −*z*+1; (iv) *x*, −*y*+1/2, *z*+1/2; (v) −*x*+1, *y*+1/2, −*z*+1/2; (vi) *x*−1, *y*, *z*; (vii) −*x*, *y*+1/2, −*z*+1/2.
